# Supplementary material for: Prevalence of ESBL, AmpC and Carbapenemase-Producing Enterobacterales Isolated from Raw Vegetables Retailed in Romania
Source: Foods. 2020 Nov 24;9(12):1726. doi: 10.3390/foods9121726 (PMC7760756; doi:10.3390/foods9121726)
Supplement: Supplementary file 1 [file foods-09-01726-s001.zip › Supplementary figure 1.docx]

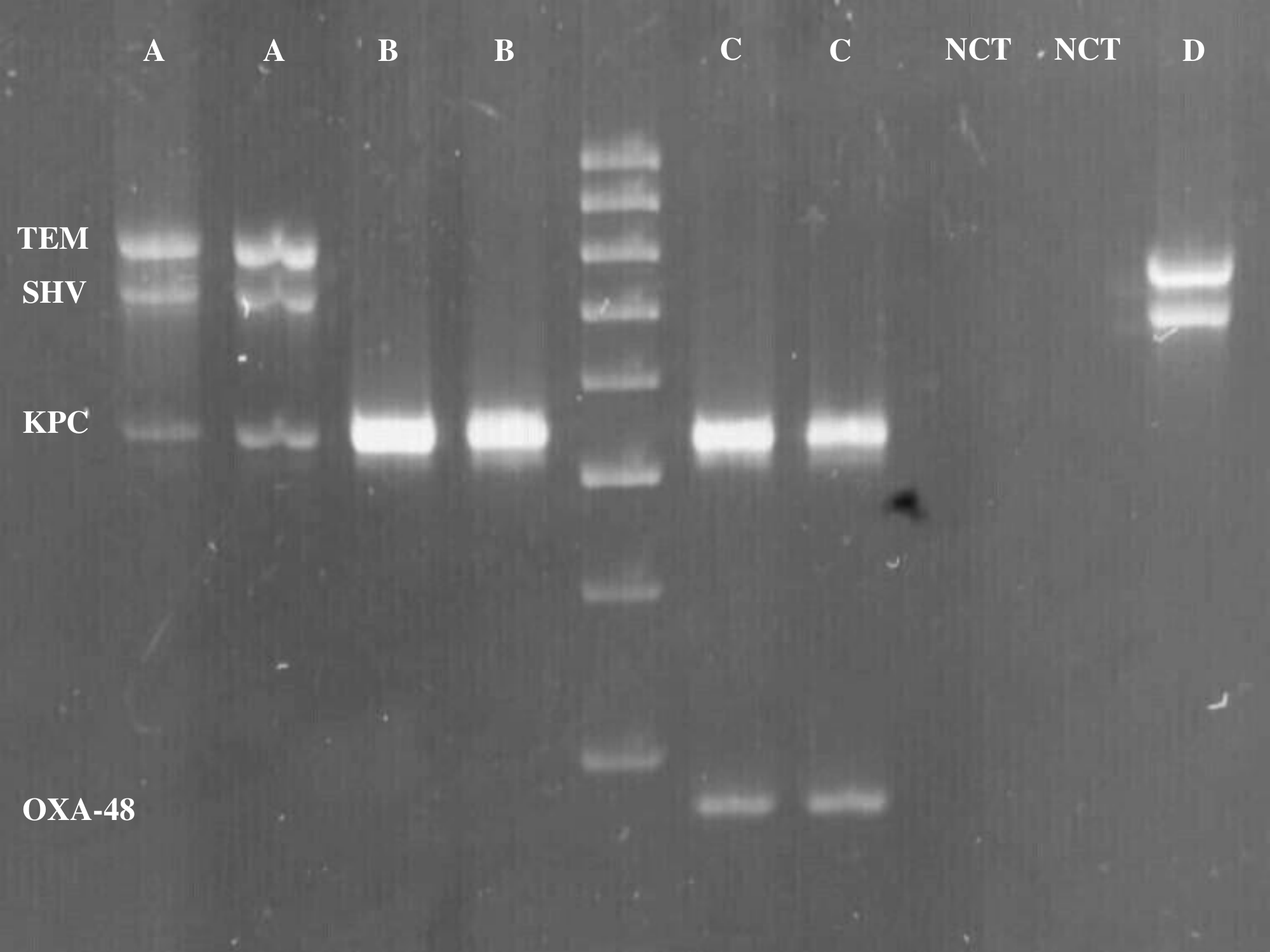


**Figure S1**. Multiplex PCR assay for the blaTEM, blaSHV, blaKPC, OXA-48 genes, amplification from control strains.

Multiplex PCR products were separated in a 1.5% agarose gel. Sample A: *K. pneumoniae* 14258; Sample B: *Pseudomonas aeruginosa* 14134; Lane 5: Molecular size marker (100 bp); Sample C: *Serratia marcescens* 13449; NCT: Negative Control; Sample D: *Serratia marcescens* 14252
