# Supplementary figures and images for: Prevalence of ESBL, AmpC and Carbapenemase-Producing Enterobacterales Isolated from Raw Vegetables Retailed in Romania
Source: Foods. 2020 Nov 24;9(12):1726. doi: 10.3390/foods9121726 (PMC7760756; doi:10.3390/foods9121726)

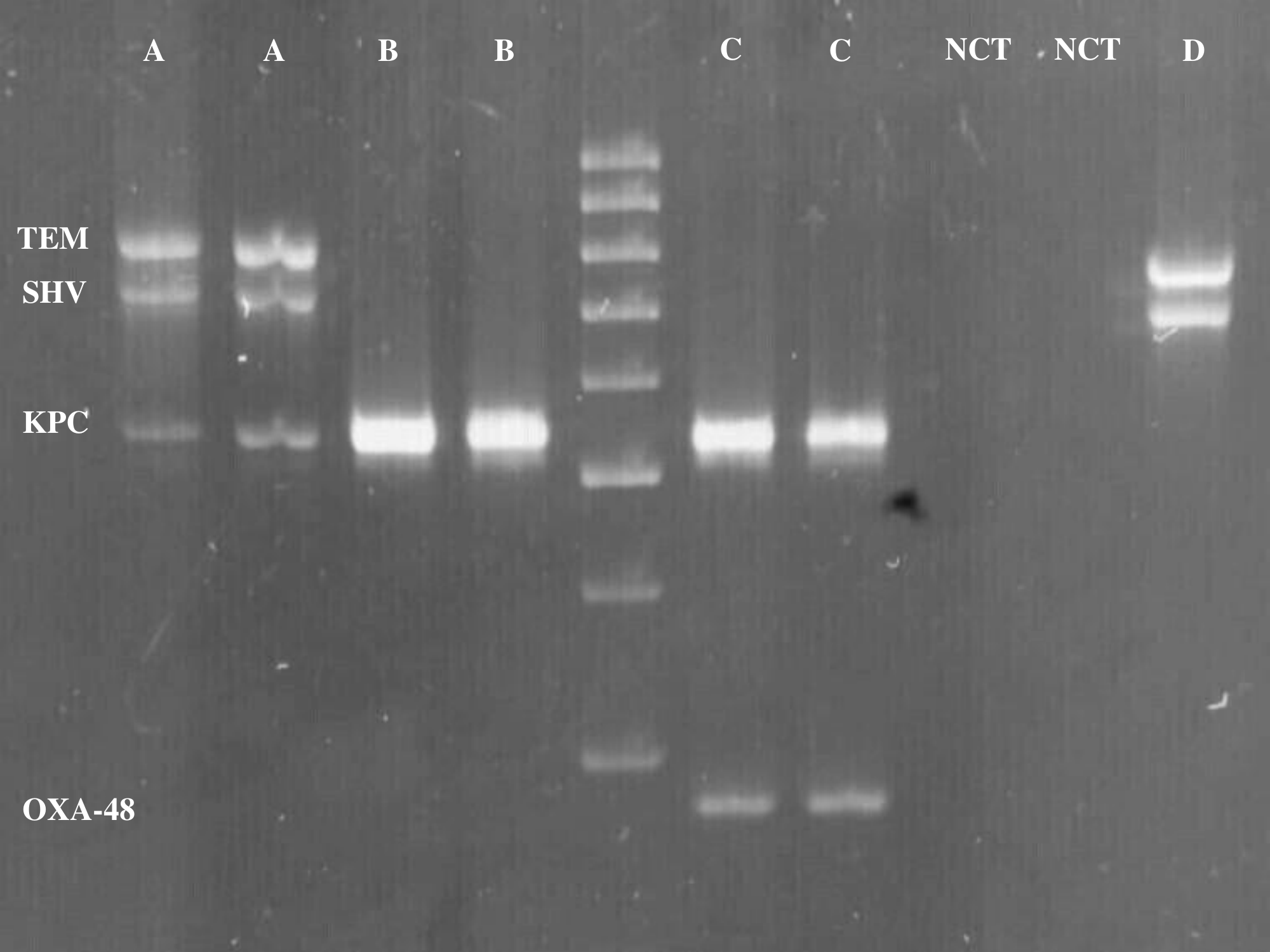

Supplement: Supplementary file 1 [file foods-09-01726-s001.zip › Supplementary Figure 1.tiff]
